# Is a Motor Criterion Essential for the Diagnosis of Clinical Huntington Disease?

**DOI:** 10.1371/currents.hd.f4c66bd51e8db11f55e1701af937a419

**Published:** 2013-04-11

**Authors:** Clement T Loy, Elizabeth A McCusker

**Affiliations:** Huntington Disease Service, Westmead Hospital, Sydney, Australia; School of Public Health, the University of Sydney, Sydney, Australia; Huntington Disease Service, Westmead Hospital, Sydney, Australia; Westmead Medical School, the University of Sydney, Sydney, Australia

## Abstract

While there has been a guideline for laboratory/genetic diagnosis of Huntington Disease (HD) since 1998, no such statement exists for the diagnosis of clinical HD. Informally, the most frequently used criteria for diagnosis of clinical HD is ‘Motor 4’ within the Unified Huntington Disease Rating Scale ’99 (motor), made when the rater is highly confident that ‘motor abnormalities observed are unequivocal signs of HD’. Recent studies involving pre-manifest individuals illustrated the shortcomings of this motor-only diagnostic approach. For instance, PREDICT-HD found cognitive changes decades before the expected date of motor diagnosis. Using a number of case studies, we highlight some of the subtleties involved in diagnosing clinical HD, in the absence of unequivocal motor signs for HD. New, broader, criteria for the diagnosis of clinical HD would be helpful in many ways. However its formulation will need to flexible rather than prescriptive, and will require extensive consultation with clinicians and families with HD.

## Introduction

While there has been a guideline for laboratory/genetic diagnosis of Huntington Disease (HD) since 1998^1^ , no such statement exists for the diagnosis of clinical HD. This partly reflects the complexity of clinical HD. HD is a multi-system disease with a continuum of manifestations, and it is diagnosed in a variety of clinical settings, with significant social and legal consequences.

Informally, the most frequently used criteria for diagnosis of clinical HD is ‘Motor 4’- a rating within the Unified Huntington Disease Rating Scale ’99 (motor)^2^ , made when the rater is highly confident that ‘motor abnormalities observed are unequivocal signs of HD’. There is no single agreed threshold in the motor score, which automatically determines whether a person qualifies as ‘Motor 4’ or not. Further judgment based on the nature of the observed motor impairment is required, and some variability between raters is likely. Nonetheless, ‘Motor 4’ usually means that chorea is present, although most HD centres would have diagnosed clinical HD without chorea for some of their patients.

Recent studies involving pre-manifest individuals have highlighted the shortcomings of this motor-only diagnostic approach. For instance, PREDICT-HD demonstrated cognitive changes decades before the expected date of motor diagnosis.^3^ In addition, over 35% of participants diagnosed with clinical HD during the PREDICT-HD study, were diagnosed with clinical HD prior to reaching the ‘Motor 4’ criteria.^4^ An alternative criteria for diagnosis of clinical HD would be helpful in many ways, but its formulation will involve much subtlety, as illustrated by the following case studies (details altered to protect privacy).

## Cases Studies

Diagrammatically, diagnosis of clinical HD can be represented by two concentric circles, divided into quadrants (Fig.1)

**Diagrammatic representation of HD:**
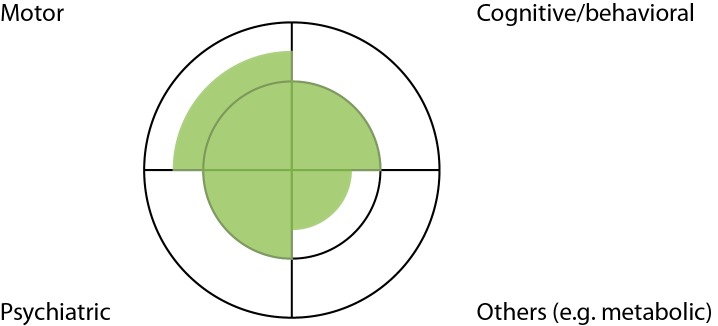
The four quadrants represent the four domains of manifestations, and the inner circle represents the thresholds for ‘clinical significance’.

The four quadrants represent the four domains of manifestations, and the inner circle represents the thresholds for ‘clinical significance’.The four quadrants represent HD manifestations involving the motor, cognitive/behavioral, psychiatric and ‘others’ (eg. metabolic/weight loss) domains. The inner circle represents thresholds for ‘clinical significance’. These thresholds are somewhat arbitrary- for instance, jerky pursuit eye movements in a HD expansion carrier is often not regarded as a sign sufficient for diagnosis of clinical HD, whilst chorea is. The shaded-in areas represent degrees of impairment along the continuum of symptoms/signs for each domain. For instance, Figure 1 represents a ‘typical’ person with HD- with chorea, enough cognitive impairment to affect work performance, some depression and weight loss. To satisfy the ‘Motor 4’ criteria, a person will need to have motor impairment beyond the inner circle/threshold in the top left quadrant.

Case 1: A 40 year old barrister has a family history of HD, involving his father who was diagnosed with HD with motor manifestations aged 55. This barrister carries the HD expansion, but has otherwise been healthy apart from moderate alcohol use. His wife has noted increasing crankiness over the past year, which he attributed to busyness at work. There has been no change in his work performance. Motor examination found jerky pursuit eye movements only. Neuropsychological examination identified mild but definite decline in cognitive function, compared to cognitive examination a year ago, in a pattern consistent with HD. He was not depressed (Fig 2).

**Case Study One:**
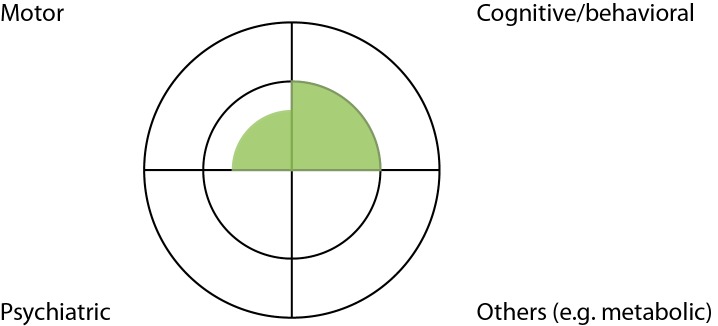

Discussion: This man did not satisfy the ‘Motor 4’ criteria for clinical HD. He had measurable cognitive decline over a year, which may prompt a diagnosis of clinical HD- but this needs to be moderated by the apparent lack of functional consequences (normal work performance according to work colleagues). Incorporation of cognitive impairment into the new diagnostic criteria raises the challenge of a suitable threshold. Functional measures (eg. inability to work in a person’s usual job) have been used as thresholds for diagnosis in other conditions. However this may represent different levels of cognitive impairment for different people when measured in absolute terms, because ability to work can depend on the nature of the occupation and social circumstances. Premature clinical diagnosis for this man could cost him his career. This man was a known expansion carrier in a stable social setting, so it was safe to stretch the process of clinical diagnosis over a longer period of time. Therefore we explained that, overall, he was still regarded as pre-manifest, but we would like to review him on a six monthly basis. A year later, his ‘crankiness’ has resolved with an improved work environment, but his neuropsychological impairment remained.

Case 2: A 23 year old man attended our clinic requesting a genetic test for HD. His mother developed clinical manifestations of HD in her 30s and was known to our Service. At the time of consultation this man was an inpatient in a mental health facility. He was admitted following a second episode of psychosis, with auditory hallucinations and some atypical features (a body dysmorphic delusion). Motor examination was normal except for mildly impaired eye movements. He was on a moderate dose of atypical anti-psychotic medication but his neurologic examination was reported to be normal prior to initiating medication. He was somewhat reluctant during neuropsychological examination and performed poorly in a range of measures, over a broader range of cognitive domains than one would expect for a typical, early, HD patient. (Fig.3)

**Case Study Two:**
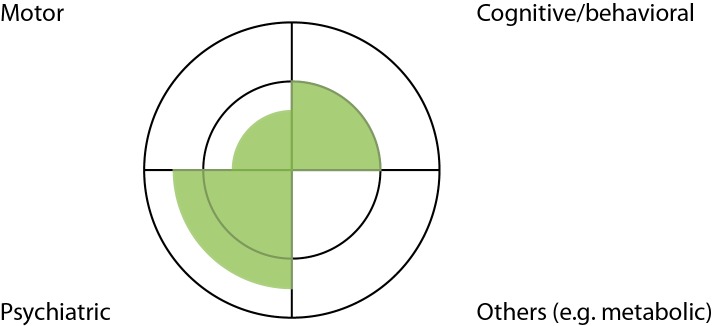

Discussion: It is possible that this man’s psychosis was part of HD, although his cognitive impairment did not lend as much support to the diagnosis of clinical HD as it would have, if the pattern had been typical for HD. This illustrates some of the difficulties in incorporating psychiatric symptoms into the diagnosis of clinical HD. Since these psychotic episodes occurred in his early twenties, they could potentially be due to schizophrenia rather than HD. If the first psychotic episode had occurred in his early 50s instead, then it would have been a little more specific for HD. This case also demonstrates the flow-on effect of the clinical diagnostic criteria, on the decision to carry out genetic testing on a pre-symptomatic basis or not. Given the uncertainty in his diagnosis, we suggested genetic testing on a pre-symptomatic, rather than diagnostic basis. The geneticist advised him to reconsider the decision to test, given his cognitive impairment. This man has an identical twin who was uncontactable at the time, which added to our hesitation to offer testing. When seen twelve months later, his psychosis had resolved, cognitive performance improved and he no longer wanted to have a genetic test.

Case 3: A 37 year old man presented with a three year history of forgetfulness and inability to cope with his work as a handyman. His mother died aged 48 with a dementia but a more detailed diagnosis was not available. He attended the clinic of an experienced neurologist during that period who noted worsening dementia of moderate severity. He also clearly documented that there has been no chorea. The patient was initially tested for mutations in the Alzheimer Disease genes, but was eventually tested for HD, about 6 months prior to presentation to our Service. He had 49/18 CAG repeats, confirming a diagnosis of HD. (Fig.4)

**Case Study Three:**
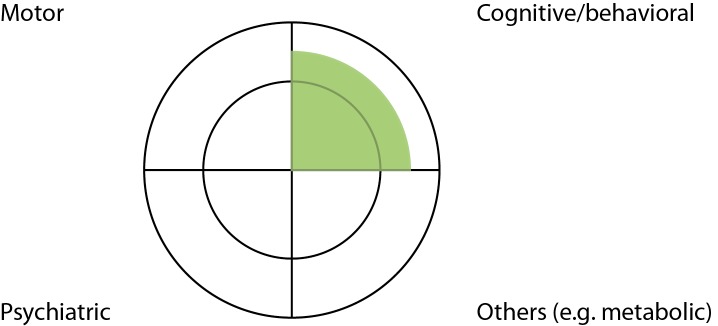

Discussion: When interviewed, this patient’s wife said she wished the diagnosis had been made earlier. It would have helped her and her young child to better understand the illness and plan for the future. New diagnostic criteria highlighting the cognitive aspects of HD, may help lead to earlier diagnosis in similar situations. Interestingly, many families encountering HD for the first time express wish for early diagnosis, while families with established family histories often prefer to delay diagnosis. Any new diagnostic criteria will need to allow some flexibility in this regard.

Case 4: This 36 year old man underwent pre-symptomatic genetic testing in his twenties and was found to carry the HD expansion. He presented with depression without suicidality, and responded well to citalopram. On examination he had mild impairment in eye movements and some possible fidgetiness in his feet only. Bedside cognitive examination was borderline normal. He spent most days playing computer games and had been unsuccessful in finding employment. He lived with his mother and would like to live in his own flat. (Fig.5)

**Case Study Four:**
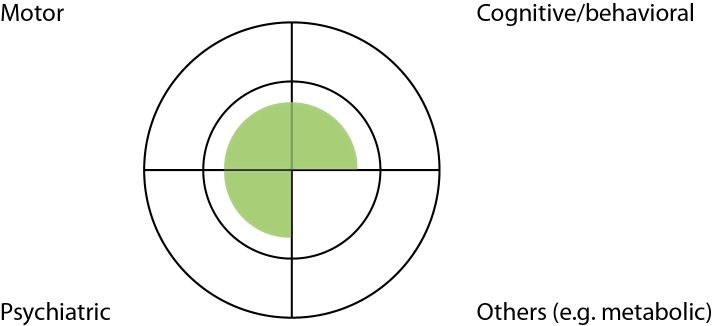

Discussion: This patient’s motor signs were not significant enough to justify a ‘Motor 4’ designation, and his cognitive performance was borderline normal. He was depressed but this may or may not be due to HD. Nonetheless he already regarded himself as symptomatic, and a clinical diagnosis would also allow him to access a Disability Support Pension, and prioritized public housing. We made the clinical diagnosis of early HD. Twelve months later, he developed a paranoid delusion which was successfully managed with a low dose of atypical psychotic.

## Concluding Remarks

Broadly speaking, the purpose of clinical diagnosis is to infer the underlying cause of a person’s symptoms and signs, in order to provide prognostic information, and to guide treatment if available. In HD the purpose of clinical diagnosis can take on a different significance. For instance, for a pre-manifest individual, the underlying cause (genetic status) may already be known, and symptomatic treatment (such as antidepressants) may already be in place. The focus of clinical diagnosis may then lie in prognostication and social/legal consequences instead.

HD is a multi-system disease with a continuum of manifestations. Brain autopsy reports of pre-manifest individuals^5^^,^ 6 suggest that changes in cellular composition/organization may begin very early in life. PREDICT-HD^3^ and TRACK-HD^7^ provide further evidence that measures of neuronal dysfunction can be detected years prior to development of definite motor signs. However, biologically, there may not be a single ‘threshold’ that we can use to define disease onset, as different biomarkers may become abnormal at different times in a person’s life. In addition, depending on the threshold chosen, sensitivity will have to be traded off against specificity for each biomarker, and not every biomarker will have the same clinical significance for different aspects of HD. Expert consensus may be the only way to determine a ‘threshold’ for diagnosis of clinical HD.

Despite this uncertainty, the notion of disease onset remains important for people with HD and their families. Likely age of disease onset is one of the most frequently asked questions by families with HD, and this information is often used for life and financial planning. Delayed diagnosis may unfairly disadvantage people with HD and cognitive-behavioral symptoms, particularly in legal proceedings. A diagnosis of clinical HD can also release superannuation, insurance, and other social benefits for people with HD.

A number of recent efforts have aimed to achieve earlier diagnosis for neurodegenerative conditions such as Alzheimer Disease^8^ and Parkinson Disease^9^ . The rationale for earlier diagnosis is to identify people likely to develop disease, and capture the therapeutic window of opportunity before neuronal damage becomes irreversible. However for HD, genetic testing already provides a highly accurate marker for future disease. While broadening the diagnostic spectrum may incidentally lead to earlier diagnosis of clinical HD in some people, earlier diagnosis is not the primary intention.

Our current ‘Motor 4’ criteria does not reflect the full spectrum of manifestations in HD. A consensus statement broadening the diagnostic spectrum will be particularly helpful for clinicians outside the HD centres. It will lend support to the diagnosis of non-motor HD by individual clinicians, if challenged in the legal setting or in the context of social benefits. This could be achieved by expert consensus and consultation with families with HD. As illustrated above, the diagnosis of clinical HD is made in a variety of settings with social and legal consequences. A flexible rather than prescriptive approach is required, and extensive consultation will be essential.

## Ethics Statement

This paper was prepared in accordance to the World Medical Association Declaration of Helsinki. The Western Sydney Local Health District Human Research Ethics Committee waived the requirement for consent as details of the cases were altered to prevent identification

## Competing Interests

The authors have declared that no competing interests exist.

## References

[1] ACMG/ASHG statement. Laboratory guidelines for Huntington disease genetic testing. The American College of Medical Genetics/American Society of Human Genetics Huntington Disease Genetic Testing Working Group. Am J Hum Genet. 1998 May;62(5):1243-7. PubMed PMID:9545416. 9545416 PMC1377103

[2] Unified Huntington's Disease Rating Scale: reliability and consistency. Huntington Study Group. Mov Disord. 1996 Mar;11(2):136-42. PubMed PMID:8684382. 8684382 10.1002/mds.870110204

[3] Paulsen JS, Langbehn DR, Stout JC, Aylward E, Ross CA, Nance M, Guttman M, Johnson S, MacDonald M, Beglinger LJ, Duff K, Kayson E, Biglan K, Shoulson I, Oakes D, Hayden M. Detection of Huntington's disease decades before diagnosis: the Predict-HD study. J Neurol Neurosurg Psychiatry. 2008 Aug;79(8):874-80. PubMed PMID:18096682. 18096682 10.1136/jnnp.2007.128728 PMC2569211

[4] Biglan KM, Zhang Y, Long J, Geschwind M, Kang G, Killoran A, Lu W, McCusker E, Mills JA, Raymond LA, Testa C, Wojcieszek J and Paulsen JS. Refining the diagnosis of Huntington Disease: The PREDICT-HD study. Front. Aging Neurosci. 2013; 5:12. 10.3389/fnagi.2013.00012 PMC3613616 23565093

[5] Albin RL, Young AB, Penney JB, Handelin B, Balfour R, Anderson KD, Markel DS, Tourtellotte WW, Reiner A. Abnormalities of striatal projection neurons and N-methyl-D-aspartate receptors in presymptomatic Huntington's disease. N Engl J Med. 1990 May 3;322(18):1293-8. PubMed PMID:1691447. 1691447 10.1056/NEJM199005033221807

[6] Gómez-Tortosa E, MacDonald ME, Friend JC, Taylor SA, Weiler LJ, Cupples LA, Srinidhi J, Gusella JF, Bird ED, Vonsattel JP, Myers RH. Quantitative neuropathological changes in presymptomatic Huntington's disease. Ann Neurol. 2001 Jan;49(1):29-34. PubMed PMID:11198293. 11198293

[7] Tabrizi SJ, Scahill RI, Durr A, Roos RA, Leavitt BR, Jones R, Landwehrmeyer GB, Fox NC, Johnson H, Hicks SL, Kennard C, Craufurd D, Frost C, Langbehn DR, Reilmann R, Stout JC. Biological and clinical changes in premanifest and early stage Huntington's disease in the TRACK-HD study: the 12-month longitudinal analysis. Lancet Neurol. 2011 Jan;10(1):31-42. PubMed PMID:21130037. 21130037 10.1016/S1474-4422(10)70276-3

[8] Sperling RA, Aisen PS, Beckett LA, Bennett DA, Craft S, Fagan AM, Iwatsubo T, Jack CR Jr, Kaye J, Montine TJ, Park DC, Reiman EM, Rowe CC, Siemers E, Stern Y, Yaffe K, Carrillo MC, Thies B, Morrison-Bogorad M, Wagster MV, Phelps CH. Toward defining the preclinical stages of Alzheimer's disease: recommendations from the National Institute on Aging-Alzheimer's Association workgroups on diagnostic guidelines for Alzheimer's disease. Alzheimers Dement. 2011 May;7(3):280-92. PubMed PMID:21514248. 21514248 10.1016/j.jalz.2011.03.003 PMC3220946

[9] Berg D. Is pre-motor diagnosis possible? The European experience. Parkinsonism Relat Disord. 2012 Jan;18 Suppl 1:S195-8. PubMed PMID:22166433. 22166433 10.1016/S1353-8020(11)70061-X

